# *N*,*N*-Dimethyl-4-[(*E*)-phenyl­imino­meth­yl]aniline

**DOI:** 10.1107/S1600536809003791

**Published:** 2009-02-11

**Authors:** Lei Zheng, Xiu-juan Yin, Cong-ling Yang, Ying Li, Shu-fan Yin

**Affiliations:** a College of Chemistry, Sichuan University, Chengdu 610064, People’s Republic of China

## Abstract

The title compound, C_15_H_16_N_2_, contains two aromatic rings linked through an imino group. The mol­ecule exhibits an *E* configuration with respect to the C=N bond. The dihedral angle between the aromatic rings is 61.96 (1)°.

## Related literature

For the physical properties and physiological activity of Schiff bases, see: Hodnett & Dunn (1970 ▶); Nyarku & Mavuso (1998 ▶); Tang & Vanslyke (1987 ▶); Yu *et al.* (2001 ▶). For related structures, see: Ahmet *et al.* (1994 ▶); Nakai *et al*. (1976 ▶); Wang & Wang (2007 ▶, 2008 ▶).
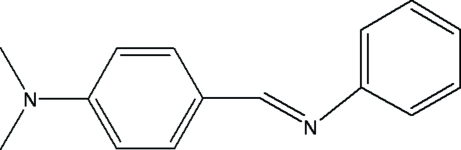


## Experimental

### 

#### Crystal data

C_15_H_16_N_2_
*M* *_r_* = 224.30
Monoclinic, 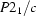

*a* = 9.441 (4) Å
*b* = 8.356 (3) Å
*c* = 17.245 (5) Å
β = 110.97 (2)°
*V* = 1270.4 (8) Å^3^
*Z* = 4
Mo *K*α radiation
μ = 0.07 mm^−1^
*T* = 292 (2) K
0.52 × 0.48 × 0.46 mm

#### Data collection

Enraf–Nonius CAD-4 diffractometer
Absorption correction: none
3023 measured reflections
2328 independent reflections
1336 reflections with *I* > 2σ(*I*)
*R*_int_ = 0.012
3 standard reflections every 200 reflections intensity decay: 1.8%

#### Refinement

*R*[*F* ^2^ > 2σ(*F* ^2^)] = 0.047
*wR*(*F* ^2^) = 0.150
*S* = 1.02
2328 reflections
157 parameters
H-atom parameters constrained
Δρ_max_ = 0.13 e Å^−3^
Δρ_min_ = −0.14 e Å^−3^

### 

Data collection: *DIFRAC* (Gabe *et al.*, 1993 ▶); cell refinement: *NRCVAX* (Gabe *et al.*, 1989 ▶); data reduction: *NRCVAX*; program(s) used to solve structure: *SHELXS97* (Sheldrick, 2008 ▶); program(s) used to refine structure: *SHELXL97* (Sheldrick, 2008 ▶); molecular graphics: *ORTEP-3 for Windows* (Farrugia,1997 ▶); software used to prepare material for publication: *SHELXL97*.

## Supplementary Material

Crystal structure: contains datablocks global, I. DOI: 10.1107/S1600536809003791/pv2134sup1.cif

Structure factors: contains datablocks I. DOI: 10.1107/S1600536809003791/pv2134Isup2.hkl

Additional supplementary materials:  crystallographic information; 3D view; checkCIF report

## Figures and Tables

**Fig. 1.**
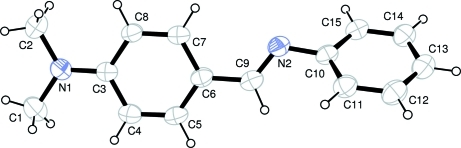
The molecular structure of (I), with displacement ellipsoids drawn at the 30% probability level.

## supplementary crystallographic information

### Comment

Schiff base, because of its unique light, electric, magnetic and other physical material properties (Tang & Vanslyke, 1987; Yu *et al.*, 2001), good coordination chemistry performance, unique anti-bacterial, anti-cancer and other physiological activities (Nyarku & Mavuso, 1998; Hodnett & Dunn, 1970), has aroused broad, systematic and in-depth theoretical and applied research. Several crystal sturctures of schiff bases, which are closely related to the title compound, have been reported (eg., Ahmet *et al.*, 1994; Nakai *et al.*, 1976; Wang & Wang, 2008; Wang & Wang, 2007). We report herein the crystal sturcture of the title schiff base, N,N-dimethyl-4-[(E)-(phenylimino)methyl]benzenamine, (I).

The molecule of the title compound (Fig. 1) adopts an E configuration probably owing to the steric effect. The C(10)–N(2)–C(9)–C(6) and C(7)–C(6)–C(9)–N(2) torsion angles are -176.70 (15) and 9.2 (3)°, respectively. There are no intermolecular hydrogen-bonding interactions in the crystal structure. The packing is essentially stabilized *via* van der Waals forces.

### Experimental

To a solution of *N*,*N*-dimethyl-4-aminobenzaldehyde (0.75 g, 5 mmol) in ethanol (10 ml) and aniline (0.91 ml, 10 mmol) were added three drops of acetic acid as a catalyst. The mixture was heated to reflux and the reaction monitored by TLC. After completion of the reaction, on cooling to room temperature, crystals were obtained. Colourless crystals suitable for X-ray analysis were obtained by slow evaporation of an ethyl acetate solution at room temperature.

### Refinement

H atoms were positioned geometrically (C—H = 0.93–0.96 Å) and included in the refinement using a riding model, with *U*_iso_(H) = 1.2*U*_eq_ (methylene C, aromatic C), *U*_iso_(H) = 1.5*U*_eq_ (methyl C).

### Crystal data

**Table:**

C_15_H_16_N_2_	*F*(000) = 480
*M**_r_* = 224.30	*D*_x_ = 1.173 Mg m^−^^3^
Monoclinic, *P*2_1_/*c*	Mo *K*α radiation, λ = 0.71073 Å
Hall symbol: -P 2ybc	Cell parameters from 20 reflections
*a* = 9.441 (4) Å	θ = 4.4–7.1°
*b* = 8.356 (3) Å	µ = 0.07 mm^−^^1^
*c* = 17.245 (5) Å	*T* = 292 K
β = 110.97 (2)°	Block, colourless
*V* = 1270.4 (8) Å^3^	0.52 × 0.48 × 0.46 mm
*Z* = 4	

### Data collection

**Table:**

Enraf–Nonius CAD-4 diffractometer	*R*_int_ = 0.012
Radiation source: fine-focus sealed tube	θ_max_ = 25.5°, θ_min_ = 2.3°
graphite	*h* = −11→11
ω/2θ scans	*k* = −10→0
3023 measured reflections	*l* = −20→10
2328 independent reflections	3 standard reflections every 200 reflections
1336 reflections with *I* > 2σ(*I*)	intensity decay: 1.8%

### Refinement

**Table:**

Refinement on *F*^2^	Secondary atom site location: difference Fourier map
Least-squares matrix: full	Hydrogen site location: inferred from neighbouring sites
*R*[*F*^2^ > 2σ(*F*^2^)] = 0.047	H-atom parameters constrained
*wR*(*F*^2^) = 0.150	*w* = 1/[σ^2^(*F*_o_^2^) + (0.0913*P*)^2^] where *P* = (*F*_o_^2^ + 2*F*_c_^2^)/3
*S* = 1.02	(Δ/σ)_max_ < 0.001
2328 reflections	Δρ_max_ = 0.13 e Å^−^^3^
157 parameters	Δρ_min_ = −0.13 e Å^−^^3^
0 restraints	Extinction correction: *SHELXL97* (Sheldrick, 2008)
Primary atom site location: structure-invariant direct methods	Extinction coefficient: 0.032 (10)

### Special details

**Table:**

Geometry. All e.s.d.'s (except the e.s.d. in the dihedral angle between two l.s. planes) are estimated using the full covariance matrix. The cell e.s.d.'s are taken into account individually in the estimation of e.s.d.'s in distances, angles and torsion angles; correlations between e.s.d.'s in cell parameters are only used when they are defined by crystal symmetry. An approximate (isotropic) treatment of cell e.s.d.'s is used for estimating e.s.d.'s involving l.s. planes.
Refinement. Refinement of *F*^2^ against ALL reflections. The weighted *R*-factor *wR* and goodness of fit *S* are based on *F*^2^, conventional *R*-factors *R* are based on *F*, with *F* set to zero for negative *F*^2^. The threshold expression of *F*^2^ > 2σ(*F*^2^) is used only for calculating *R*-factors(gt) *etc*. and is not relevant to the choice of reflections for refinement. *R*-factors based on *F*^2^ are statistically about twice as large as those based on *F*, and *R*- factors based on ALL data will be even larger.

### Fractional atomic coordinates and isotropic or equivalent isotropic displacement parameters (Å^2^)

**Table:**

	*x*	*y*	*z*	*U*_iso_*/*U*_eq_	
N1	0.30901 (17)	1.50607 (18)	−0.07311 (10)	0.0722 (5)	
N2	0.28787 (18)	0.86291 (19)	0.13032 (9)	0.0668 (5)	
C1	0.3837 (2)	1.4806 (2)	−0.13194 (12)	0.0815 (6)	
H1A	0.4831	1.4372	−0.1037	0.122*	
H1B	0.3924	1.5807	−0.1572	0.122*	
H1C	0.3254	1.4071	−0.1740	0.122*	
C2	0.2456 (3)	1.6630 (2)	−0.07117 (15)	0.0907 (7)	
H2A	0.1369	1.6571	−0.0944	0.136*	
H2B	0.2810	1.7363	−0.1031	0.136*	
H2C	0.2768	1.6997	−0.0148	0.136*	
C3	0.28724 (19)	1.3833 (2)	−0.02654 (11)	0.0587 (5)	
C4	0.2055 (2)	1.4036 (2)	0.02656 (12)	0.0687 (5)	
H4	0.1636	1.5029	0.0300	0.082*	
C5	0.1867 (2)	1.2792 (2)	0.07319 (12)	0.0686 (5)	
H5	0.1329	1.2965	0.1082	0.082*	
C6	0.2449 (2)	1.1275 (2)	0.07036 (11)	0.0616 (5)	
C7	0.3258 (2)	1.1071 (2)	0.01746 (10)	0.0636 (5)	
H7	0.3669	1.0073	0.0142	0.076*	
C8	0.3462 (2)	1.2297 (2)	−0.02946 (11)	0.0625 (5)	
H8	0.4004	1.2115	−0.0643	0.075*	
C9	0.2222 (2)	0.9985 (2)	0.12090 (11)	0.0681 (5)	
H9	0.1550	1.0160	0.1484	0.082*	
C10	0.2496 (2)	0.7447 (2)	0.17785 (11)	0.0619 (5)	
C11	0.1006 (2)	0.7114 (2)	0.16925 (12)	0.0715 (6)	
H11	0.0217	0.7702	0.1321	0.086*	
C12	0.0694 (2)	0.5918 (3)	0.21553 (13)	0.0795 (6)	
H12	−0.0307	0.5700	0.2093	0.095*	
C13	0.1848 (3)	0.5041 (2)	0.27093 (13)	0.0796 (6)	
H13	0.1629	0.4243	0.3025	0.096*	
C14	0.3326 (2)	0.5352 (2)	0.27936 (12)	0.0744 (6)	
H14	0.4110	0.4757	0.3165	0.089*	
C15	0.3645 (2)	0.6536 (2)	0.23315 (11)	0.0682 (5)	
H15	0.4648	0.6733	0.2389	0.082*	

### Atomic displacement parameters (Å^2^)

**Table:**

	*U*^11^	*U*^22^	*U*^33^	*U*^12^	*U*^13^	*U*^23^
N1	0.0775 (11)	0.0677 (10)	0.0723 (11)	0.0087 (8)	0.0278 (9)	0.0044 (8)
N2	0.0681 (10)	0.0769 (11)	0.0590 (9)	0.0019 (8)	0.0270 (8)	−0.0015 (8)
C1	0.0882 (14)	0.0911 (14)	0.0695 (12)	−0.0005 (11)	0.0338 (11)	0.0092 (11)
C2	0.0945 (16)	0.0719 (13)	0.1018 (16)	0.0086 (11)	0.0305 (14)	0.0059 (12)
C3	0.0538 (10)	0.0656 (11)	0.0530 (10)	−0.0001 (8)	0.0146 (8)	−0.0077 (9)
C4	0.0696 (12)	0.0667 (11)	0.0716 (12)	0.0094 (9)	0.0276 (10)	−0.0083 (10)
C5	0.0684 (12)	0.0816 (14)	0.0637 (11)	0.0060 (10)	0.0333 (10)	−0.0109 (10)
C6	0.0620 (11)	0.0713 (12)	0.0535 (10)	0.0026 (9)	0.0231 (9)	−0.0057 (9)
C7	0.0682 (12)	0.0637 (11)	0.0630 (11)	0.0070 (9)	0.0285 (10)	−0.0066 (9)
C8	0.0669 (12)	0.0682 (12)	0.0580 (11)	0.0063 (9)	0.0290 (9)	−0.0048 (9)
C9	0.0657 (12)	0.0836 (14)	0.0594 (11)	0.0019 (10)	0.0278 (9)	−0.0064 (10)
C10	0.0642 (12)	0.0724 (11)	0.0528 (10)	−0.0030 (10)	0.0255 (9)	−0.0095 (9)
C11	0.0607 (12)	0.0852 (14)	0.0656 (12)	−0.0022 (10)	0.0191 (10)	−0.0030 (11)
C12	0.0657 (12)	0.0961 (15)	0.0779 (13)	−0.0151 (11)	0.0271 (11)	−0.0076 (12)
C13	0.0881 (15)	0.0816 (14)	0.0746 (13)	−0.0110 (12)	0.0358 (12)	−0.0003 (11)
C14	0.0770 (13)	0.0800 (13)	0.0680 (12)	0.0054 (11)	0.0281 (10)	0.0050 (11)
C15	0.0619 (12)	0.0796 (12)	0.0680 (12)	0.0011 (10)	0.0291 (10)	−0.0036 (11)

### Geometric parameters (Å, °)

**Table:**

N1—C3	1.363 (2)	C6—C7	1.394 (2)
N1—C1	1.444 (2)	C6—C9	1.449 (3)
N1—C2	1.447 (2)	C7—C8	1.361 (2)
N2—C9	1.274 (2)	C7—H7	0.9300
N2—C10	1.411 (2)	C8—H8	0.9300
C1—H1A	0.9600	C9—H9	0.9300
C1—H1B	0.9600	C10—C15	1.388 (3)
C1—H1C	0.9600	C10—C11	1.388 (3)
C2—H2A	0.9600	C11—C12	1.375 (3)
C2—H2B	0.9600	C11—H11	0.9300
C2—H2C	0.9600	C12—C13	1.375 (3)
C3—C4	1.403 (2)	C12—H12	0.9300
C3—C8	1.407 (2)	C13—C14	1.375 (3)
C4—C5	1.364 (2)	C13—H13	0.9300
C4—H4	0.9300	C14—C15	1.370 (3)
C5—C6	1.389 (2)	C14—H14	0.9300
C5—H5	0.9300	C15—H15	0.9300
			
C3—N1—C1	121.18 (15)	C8—C7—C6	121.63 (16)
C3—N1—C2	121.17 (16)	C8—C7—H7	119.2
C1—N1—C2	117.39 (17)	C6—C7—H7	119.2
C9—N2—C10	118.88 (16)	C7—C8—C3	121.55 (16)
N1—C1—H1A	109.5	C7—C8—H8	119.2
N1—C1—H1B	109.5	C3—C8—H8	119.2
H1A—C1—H1B	109.5	N2—C9—C6	124.65 (17)
N1—C1—H1C	109.5	N2—C9—H9	117.7
H1A—C1—H1C	109.5	C6—C9—H9	117.7
H1B—C1—H1C	109.5	C15—C10—C11	118.49 (18)
N1—C2—H2A	109.5	C15—C10—N2	118.89 (16)
N1—C2—H2B	109.5	C11—C10—N2	122.57 (18)
H2A—C2—H2B	109.5	C12—C11—C10	120.17 (19)
N1—C2—H2C	109.5	C12—C11—H11	119.9
H2A—C2—H2C	109.5	C10—C11—H11	119.9
H2B—C2—H2C	109.5	C13—C12—C11	120.63 (19)
N1—C3—C4	121.86 (16)	C13—C12—H12	119.7
N1—C3—C8	121.37 (16)	C11—C12—H12	119.7
C4—C3—C8	116.77 (17)	C12—C13—C14	119.64 (19)
C5—C4—C3	120.70 (16)	C12—C13—H13	120.2
C5—C4—H4	119.7	C14—C13—H13	120.2
C3—C4—H4	119.7	C15—C14—C13	120.05 (19)
C4—C5—C6	122.56 (16)	C15—C14—H14	120.0
C4—C5—H5	118.7	C13—C14—H14	120.0
C6—C5—H5	118.7	C14—C15—C10	121.00 (18)
C5—C6—C7	116.79 (17)	C14—C15—H15	119.5
C5—C6—C9	120.79 (17)	C10—C15—H15	119.5
C7—C6—C9	122.42 (17)		
			
C1—N1—C3—C4	−175.41 (16)	C10—N2—C9—C6	−176.70 (15)
C2—N1—C3—C4	−1.3 (3)	C5—C6—C9—N2	−170.32 (18)
C1—N1—C3—C8	4.7 (3)	C7—C6—C9—N2	9.2 (3)
C2—N1—C3—C8	178.74 (17)	C9—N2—C10—C15	−137.72 (18)
N1—C3—C4—C5	−179.28 (17)	C9—N2—C10—C11	44.8 (2)
C8—C3—C4—C5	0.6 (3)	C15—C10—C11—C12	0.7 (3)
C3—C4—C5—C6	−0.7 (3)	N2—C10—C11—C12	178.17 (16)
C4—C5—C6—C7	0.5 (3)	C10—C11—C12—C13	0.2 (3)
C4—C5—C6—C9	−179.94 (17)	C11—C12—C13—C14	−0.8 (3)
C5—C6—C7—C8	−0.3 (3)	C12—C13—C14—C15	0.4 (3)
C9—C6—C7—C8	−179.89 (17)	C13—C14—C15—C10	0.5 (3)
C6—C7—C8—C3	0.4 (3)	C11—C10—C15—C14	−1.0 (3)
N1—C3—C8—C7	179.43 (16)	N2—C10—C15—C14	−178.61 (16)
C4—C3—C8—C7	−0.5 (3)		
